# White-Light GaN-μLEDs Employing Green/Red Perovskite Quantum Dots as Color Converters for Visible Light Communication

**DOI:** 10.3390/nano12040627

**Published:** 2022-02-13

**Authors:** Xiaoyan Liu, Langyi Tao, Shiliang Mei, Zhongjie Cui, Daqi Shen, Zhengxuan Sheng, Jinghao Yu, Pengfei Ye, Ting Zhi, Tao Tao, Lei Wang, Ruiqian Guo, Pengfei Tian

**Affiliations:** 1 College of Integrated Circuit Science and Engineering, and National and Local Joint Engineering Laboratory for RF Integration and Micro-Packaging Technologies, Nanjing University of Posts and Telecommunications, Nanjing 210023, China; xiaoyanliu@njupt.edu.cn (X.L.); b18020227@njupt.edu.cn (L.T.); b18020308@njupt.edu.cn (D.S.); b18012128@njupt.edu.cn (Z.S.); 1020021023@njupt.edu.cn (J.Y.); 1320027525@njupt.edu.cn (P.Y.); zhit@njupt.edu.cn (T.Z.); 2 Institute for Electric Light Sources, School of Information Science and Technology, Fudan University, Shanghai 200433, China; meishiliang@fudan.edu.cn (S.M.); 19110720020@fudan.edu.cn (Z.C.); 3 Key Laboratory of Advanced Photonic and Electronic Materials, School of Electronic Science and Engineering, Nanjing University, Nanjing 210046, China; ttao@nju.edu.cn

**Keywords:** white-light GaN-based μLEDs, perovskite quantum dots, visible light communication, solid-state lighting

## Abstract

GaN-based μLEDs with superior properties have enabled outstanding achievements in emerging micro-display, high-quality illumination, and communication applications, especially white-light visible light communication (WL-VLC). WL-VLC systems can simultaneously provide white-light solid-state lighting (SSL) while realizing high-speed wireless optical communication. However, the bandwidth of conventional white-light LEDs is limited by the long-lifetime yellow yttrium aluminum garnet (YAG) phosphor, which restricts the available communication performance. In this paper, white-light GaN-μLEDs combining blue InGaN-μLEDs with green/red perovskite quantum dots (PQDs) are proposed and experimentally demonstrated. Green PQDs (G-PQDs) and red PQDs (R-PQDs) with narrow emission spectrum and short fluorescence lifetime as color converters instead of the conventional slow-response YAG phosphor are mixed with high-bandwidth blue InGaN-μLEDs to generate white light. The communication and illumination performances of the WL-VLC system based on the white-light GaN-based μLEDs are systematically investigated. The VLC properties of monochromatic light (green/red) from G-PQDs or R-PQDs are studied in order to optimize the performance of the white light. The modulation bandwidths of blue InGaN-μLEDs, G-PQDs, and R-PQDs are up to 162 MHz, 64 MHz, and 90 MHz respectively. Furthermore, the white-light bandwidth of 57.5 MHz and the Commission Internationale de L’Eclairage (CIE) of (0.3327, 0.3114) for the WL-VLC system are achieved successfully. These results demonstrate the great potential and the direction of the white-light GaN-μLEDs with PQDs as color converters to be applied for VLC and SSL simultaneously. Meanwhile, these results contribute to the implementation of full-color micro-displays based on μLEDs with high-quality PQDs as color-conversion materials.

## 1. Introduction

Group III-nitride semiconductors are among the most popular wide-bandgap semiconductors owing to their superior advantages of high electron mobility, wide bandgap, high stability, and high breakdown voltage [1,2,3]. The wide direct bandgap, ranging from deep ultraviolet (~6.2 eV) to near-infrared (~0.7 eV), can be tuned to span the entire UV and visible spectrum. Therefore, group III-nitride semiconductors have gathered enormous attention and undergone rapid development in varied applications of optoelectronics and power electronics such as light-emitting diodes (LEDs), laser diodes (LDs), photodetectors, high-electron-mobility transistors (HEMTs), and so forth [4,5,6,7,8]. Among them, GaN-based LEDs have become some of the most mature and influential optoelectronic devices since they were first demonstrated in the 1990s [9]. They have been widely used in solid-state lighting (SSL), outdoor and indoor displays, and backlight sources owing to their high efficiency and endurance, high cost efficiency, low power consumption, etc. [10,11,12]. More recently, micron-scale LEDs sized from 1 μm to 100 μm, known as micro-LEDs (μLEDs), have become a favorite topic in academia and industry. In contrast with broad-area LEDs, GaN-based μLEDs possess excellent electronic and optical properties including lower resistance-capacitance (RC) delay, high bandwidth, high efficiency, high brightness, and high contrast. Thanks to these outstanding characteristics, GaN-based µLEDs have seen development beyond SSL, and are used in cutting-edge applications such as micro-displays, augmented reality and virtual reality, optogenetics, and visible light communications (VLC) [13,14,15]. In 2010, the GaN-based μLED was first employed in VLC by Martin D. Dawson from the University of Strathclyde [16]. Since then, μLEDs have attracted extensive attention and achieved dramatic advances in the VLC field [17,18,19,20,21,22].

As a promising candidate for next-generation wireless communication technology, VLC—particularly white-light visible light communication (WL-VLC), combining SSL and optical wireless communication together—has been pursued by the academic community and industry. Utilizing light as a data-transmission carrier, VLC possesses the advantages of a broad frequency range, high data rate, greater security, higher energy efficiency [23], and lack of radio frequency (RF) interferences. Due to the limited bandwidth and the crowded communication window of existing RF and microwave wireless communication technologies, a great deal of effort has been made to prove that VLC has an enormous potential to fulfill the surging demand for the informatization exchange of modern society. Commonly, the conventional white-light LEDs used for SSL consist of the blue light from a broad-area LED and the yellow light from YAG:Ce^3+^ phosphor. Nevertheless, the YAG:Ce^3+^ phosphor suffers from long photoluminescence (PL) lifetime and low modulation bandwidth (less than 20 MHz), showing limited capability for VLC [24,25]. With the peculiarities of high PL quantum yield, short PL lifetime, and narrow emission bandwidth, perovskite quantum dots (PQDs) present an appealing opportunity in next-generation lighting sources for both illumination and communication [26,27,28,29,30,31]. Dursun et al. utilized LDs to excite CsPbBr_3_ perovskite nanocrystals and a conventional red phosphor to generate the white light for VLC [32]. Liang et al. synthesized the CsPbBr_3_ liquid perovskite quantum dots which were applied in an LD-based white-light VLC system [33]. The maximum transmitted data rates at a receivable bit error rate (BER) of two systems were up to 2 Gbps and 1 Gbps, respectively [32,33]. The LD-based VLC systems have outstanding performances in wireless communication, but are poor in the SSL field owing to problems such as limited etendue, detriment to human eyes, and speckle noise [34]. Therefore, light sources (such as LED) that are more acceptable for human eyes and the environment in the long term are desired. In contrast to broad-area LED, μLEDs with a feature size of tens of micrometers enjoy excellent intrinsic properties such as larger current saturation density and higher modulation bandwidth [19,35,36], revealing their enormous potential in both VLC and SSL fields. Leitão et al. set up a VLC system based on a CsPb_4_Br_6_ matrix with CsPbBr_3_ QDs and an InGaN µLED, with a corresponding modulation bandwidth of up to 24.6 MHz [37]. In our previous report, a high-bandwidth white-light system was proposed which consisted of yellow-emitting CsPbBr_1.8_I_1.2_ perovskite quantum dots and a blue GaN-based μLED. The system has presented a high bandwidth of up to 85 MHz and a maximum data rate of 300 Mbps [38]. Although some progress has been made in WL-VLC, there is a trade-off between illumination performance and communication capacity, so it is important to balance high-quality lighting and high-speed communication. Therefore, many efforts need to be made to pursue high-quality white-light systems that can simultaneously implement high-quality SSL and high-capacity VLC dual-function applications. Several types of color converters have been explored for use as color-conversion layers to generate high-quality white light while maintaining high-bandwidth communication performance.

Hence, in this paper, green PQDs (G-PQDs) and red PQDs (R-PQDs) are employed as color converters, which are excited by blue InGaN-μLEDs to produce the white light for SSL and VLC. The characteristics of monochromatic light (red/green) from G-PQDs and R-PQDs are studied to obtain high-quality illumination while maintaining high modulation bandwidth. The SSL properties and VLC performances of the obtained white-light system are further investigated in detail. In this study, the maximum −3 dB modulation bandwidths of monochromatic light and white light were up to ~64 MHz (green-light), ~90 MHz (red-light), and 57.5 MHz (white-light), respectively. Moreover, the white light generated by blue InGaN-μLEDs, G-PQDs, and R-PQDs possessed a Commission Internationale de L’Eclairage (CIE) of (0.3327, 0.3114) and the color temperature of 5474 K.

## 2. Experimental Methods

### 2.1. Fabrication of InGaN-μLED Device

Blue-emitting InGaN-μLEDs were fabricated from a commercial GaN epitaxy wafer grown on c-plane sapphire substrates. An n-GaN layer, an InGaN/GaN multiple quantum well (MQW) layer, an AlGaN electron blocking layer, and a p-GaN layer were deposited on the patterned sapphire substrates through metal-organic chemical vapor deposition. 

**Figure 1:**
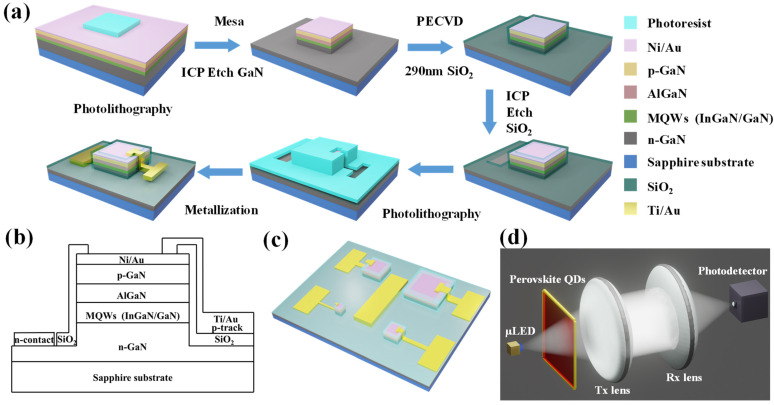
(**a**) The schematic diagram of the typical fabrication process and (**b**) the epitaxial structure of square InGaN-μLED. (**c**) The 3D schematic diagram of square InGaN-μLED arrays with different sizes. (**d**) Schematic diagram of WL-VLC system based on the white-light GaN-based μLED with PQDs as color converters.

A series of fabrication processes including photolithography, wet and dry etching, film deposition, lift-off, etc. were performed in a cleanroom. The following briefly describes the typical fabrication processes of the InGaN-μLED, which is shown in Figure 1a. The Ni/Au (10 nm/25 nm) metal was deposited as a current spreading layer by magnetron sputtering or electron-beam evaporation before fabrication of the InGaN-μLED device. Next, utilizing inductively coupled plasma etching (ICP), the epitaxial structure was etched to the n-GaN layer, and the μLED mesa was manufactured. This was followed by rapid thermal annealing in nitrogen at 500 °C to form an ohmic contact. Then, a 290 nm SiO_2_ layer was deposited as a standard isolation layer by plasma-enhanced chemical vapor deposition (PECVD). Lithographic patterning and wet/dry etching processes were used to etch SiO_2_ to open apertures on the μLED mesas and the n-contact area to deposit metal. Finally, via photolithography and metallization, the Ti/Au (50 nm/200 nm) metal was deposited as n-track and p-track. The fabrication details can be found in previous work [19,35,38,39,40,41,42]. In this way, the μLED arrays with square and circular mesas and various sizes were fabricated successfully. The diameters for circular μLED pixels were 40, 60, and 100 μm. For square μLED pixels, the side lengths were 40, 60, and 80 μm. The 2D epitaxial structure of a single square InGaN-μLED is shown in Figure 1b. Figure 1c presents a 3D schematic diagram of the InGaN-μLED arrays with square pixels.

### 2.2. Fabrication of GaN-Based White-Light μLEDs

GaN-based white-light micro-LEDs were fabricated by employing blue InGaN-μLEDs as light sources and PQDs as the color converters. PQDs were synthesized through a modified hot-injection method based on a previous study [43]. Under a nitrogen atmosphere and at a reaction temperature of 150 °C, Cs-oleate solution was quickly injected into a mixture of octadecene, oleylamine, oleic acid, and PbX_2_ (X = Br or I). The as-prepared green CsPbBr_3_ QDs (G-PQDs) were extracted after discarding the supernatant. The red CsPb(Br/I)_3_ QDs (R-PQDs) were fabricated in the same way. The fabricated G-PQDs and R-PQDs were encapsulated in epoxy resin, which isolated them from oxygen and moisture. The detailed synthesis processes can be found in our previous study [38]. The prepared PQD films acting as color converters were overlaid on the blue-emitting InGaN-μLEDs, and the white light was obtained under excitation of the μLEDs. Then, the InGaN-μLED chip covered with PQDs was bonded to a printed circuit board. Finally, the hybrid white-light GaN-based devices were implemented.

### 2.3. Construction of WL-VLC System

As presented in Figure 1d, the main part of the VLC system consisted of blue InGaN-μLED, G-PQDs and R-PQDs, a transmitter lens (Tx lens), a receiver lens (Rx lens), and a photodetector. The blue-emitting μLEDs were employed as light sources to excite different PQDs which could adjust the color component of light required by the VLC system. Using a non-return-to-zero on–off key (NRZ-OOK) modulation scheme, the signals were modulated to the μLEDs by the bias tee. The modulated light propagated through the free-space transmission link. Color converters were put into the light path to fulfill the experimental requirements. The Tx lens and Rx lens were used to collimate and focus the modulated light generated from the light-emitter. The light was received and recorded by a photodetector, and then the light signals were converted to electric signals for further analysis of the frequency response, BER, and eye diagrams. Based on the above VLC link, a network analyzer (Agilent, N5225A, 10 MHz–50 GHz) was used to measure the frequency response to obtain the modulation bandwidth under different currents. The BER was measured by an error-detector module built into the signal quality analyzer (Anritsu, MP1800A, 0.1–14 GHz). A wide-bandwidth oscilloscope (Agilent 86100A, 14 GHz) was used to capture eye diagrams. All measurements were carried out at ambient temperature in air.

## 3. Results and Discussion

### 3.1. Electrical and Optical Properties of InGaN-μLEDs

**Figure 2:**
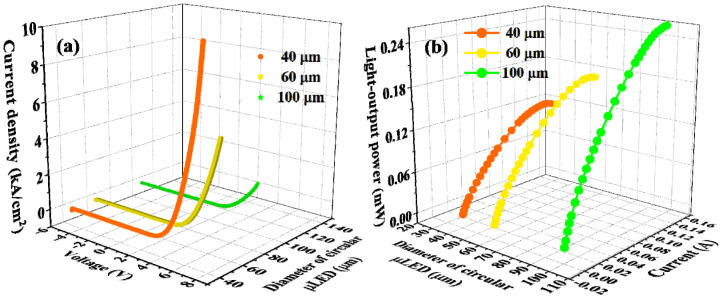
(**a**) J–V and (**b**) L–I characteristics of circular InGaN-μLEDs with different diameters.

We studied the electrical and optical properties of the fabricated InGaN-μLEDs before they were embedded into the system. The relationship between current density and bias voltage (J–V) and that between light output power and injection current (L–I) of circular μLEDs with different diameters are illustrated in Figure 2. The figure indicates a dependent relationship between output performance and device scale. That is, the smaller the μLEDs, the higher the current density that the μLEDs could sustain, and a lower light output that the μLEDs could produce. These results were in accordance with the J–V and L–I characteristics of the InGaN-μLEDs with square pixels, as shown in Figure S1. Therefore, there was a trade-off between modulation bandwidth and light-output power, which will be discussed in the following section.

**Figure 3:**
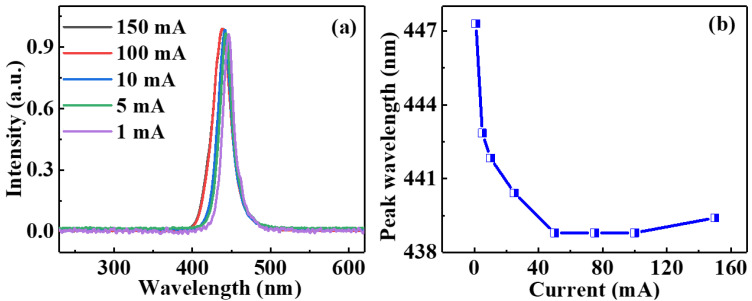
(**a**) The normalized EL spectra of circular μLED with a diameter of 80 μm under different currents. (**b**) Peak wavelength extracted from the EL spectra at different injection currents.

Figure 3 demonstrates the normalized electroluminescence (EL) spectra of the circular InGaN-μLEDs, taking 80 μm μLED as an example. In Figure 3a, with increasing injection current, the peak wavelength of the EL spectrum moved to the shorter wavelength direction and the full width at half maximum (FWHM) increased, then both kept saturation. Both the movement of peak wavelength and the increase of FWHM were on a small scale, ensuring good monochromaticity for SSL. Figure 3b shows the peak wavelength as a function of the injection current, which was extracted from Figure 3a. The blueshift of the emission wavelength observed is attributed to the carrier-screening effect of the quantum-confined Stark effect and/or the band-filling effect [44], which degrades the performance of μLEDs (e.g., aging, reliability, and modulation bandwidth) [45,46]. As a result, to obtain optimal modulation bandwidth and light-output power, the appropriate working current was chosen to drive the μLEDs. For InGaN-μLEDs with square pixels, the EL spectra were similar to those of InGaN-μLEDs with circular pixels, as shown in Figure S2.

### 3.2. Frequency Responses of InGaN-μLEDs

**Figure 4:**
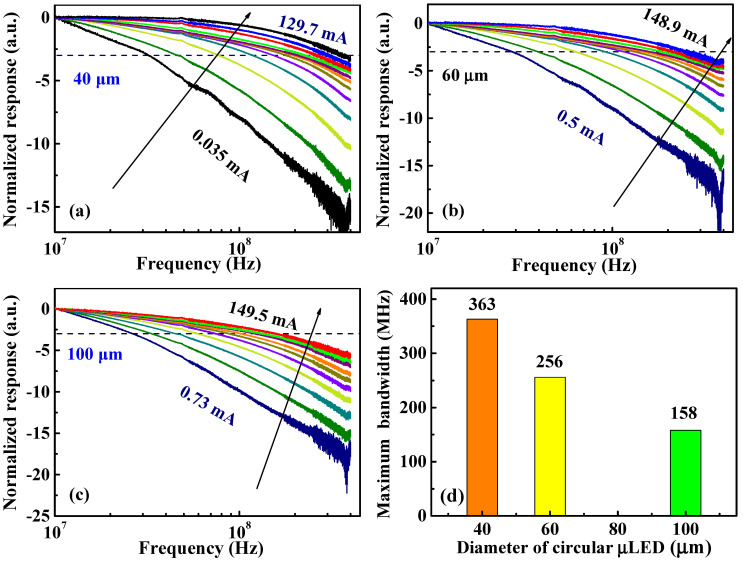
Frequency responses of the circular InGaN-μLEDs with the diameters of (**a**) 40 μm, (**b**) 60 μm, and (**c**) 100 μm under different injection currents. The –3 dB modulation bandwidth is marked by a dashed line. (**d**) The maximum bandwidths of InGaN-μLEDs with different sizes.

To study the modulation bandwidth of the InGaN-μLED for high-speed VLC, we evaluated the normalized electrical-to-optical frequency response of the circular μLEDs with different sizes under the injection current varying roughly in the region from approximately 0 mA (~hundreds of nA) to 150 mA (Figure 4a–c). As shown in Figure 4a, with the increase of injection current, the modulation bandwidth first increased then saturated for the circular μLED with a diameter of 40 μm. In Figure 4b,c, the characteristics of the modulation bandwidth versus injection current for circular μLEDs with diameters of 60 μm and 100 μm were consistent with that of 40 μm μLEDs (Figure 4a). The higher bandwidth was obtained at a higher current, which can be attributed to the reduced differential carrier lifetime at higher injection currents [42]. The bandwidth saturation might be attributed to the impedance mismatch between the μLED and the package [39]. The highest bandwidths of μLEDs with different sizes are shown in Figure 4d. It is evident that attainable modulation bandwidth increased as the size of the µLEDs decreased, further indicating that InGaN-μLEDs have greater potential in the field of VLC than broad-area LEDs. For InGaN-μLEDs with square pixels, the frequency response of the μLED with a side length of 80 μm has been studied in previous work [38,39]. The change trend of bandwidth versus current was consistent with that of the e circular InGaN-μLEDs.

Although smaller μLEDs possessed higher bandwidth (Figure 4), the output power was decreased owing to the smaller mesa area (Figure 2), leading to a higher BER that limits communication speed and transmission distance. In this work, the InGaN-μLEDs as light sources excited PQDs and mixed them to generate white light for SSL and VLC, so a higher light-output power was desired. Comparing Figure 2b with Figure S1b, it is evident that the square InGaN-μLED with the side length of 80 μm had the maximum light-output power (up to 1.3 mW) among the samples. In addition, note that in Figure S3, the square μLED with the side length of 80 μm presented a maximum bandwidth bandwidth of about 162 MHz. Therefore, to balance the bandwidth of the μLED for VLC and the light-output performance for the SSL, the square InGaN-μLED with the side length of 80 μm was applied for the WL-VLC system.

### 3.3. Visible Light Communication and Solid-State Lighting

**Figure 5:**
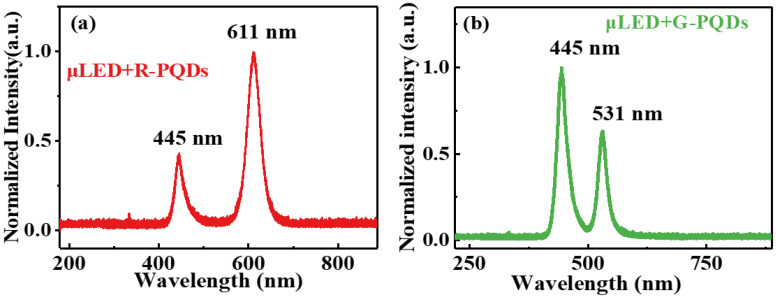
PL spectra of light generated by μLEDs exciting two kinds of luminescent materials: (**a**) R-PQDs (**b**) and G-PQDs.

Before the construction of the white-light system, two kinds of color-conversion materials (R-PQDs and G-PQDs) were utilized separately to generate monochromatic light for communication, and the properties of both PQDs are discussed in the following. Figure 5a presents the spectrum of R-PQDs under excitation of the μLED, where one peak wavelength was at 445 nm from the μLED and another peak wavelength was at 611 nm from R-PQDs. Likewise, the spectrum of G-PQDs excited by μLED is shown in Figure 5b, with two peak wavelengths representing μLED and G-PQDs at 445 nm and 531 nm, respectively. The FWHMs of the R-PQDs and G-PQDs were 34 nm and 21 nm, manifesting the outstanding narrow emission of PQDs.

**Figure 6:**
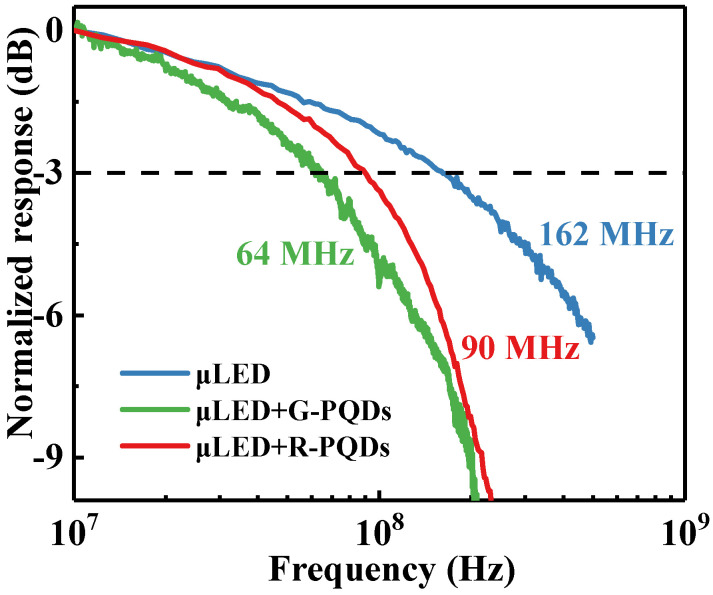
Frequency responses of the square μLED, R-PQDs, and G-PQDs. The –3 dB modulation bandwidth is marked by a dashed line.

The normalized responses of R-PQDs and G-PQDs excited by the blue InGaN-μLEDs were measured, which are exhibited in Figure 6. The bandwidth of the square InGaN-μLED with the size of 80 μm was up to ~162 MHz, and under the excitation of the μLED, the bandwidths of R-PQDs (μLED + R-PQDs) and G-PQDs (μLED + G-PQDs) were ~90 MHz and ~64 MHz, respectively. Meanwhile, the R-PQDs showed better stability and higher bandwidth than the G-PQDs as the curve declined more slowly and smoothly. The superiority of PQDs can be supported by drawing a comparison with the slow frequency responses of conventional YAG:Ce^3+^ phosphors (2.5 MHz) [25].

**Figure 7:**
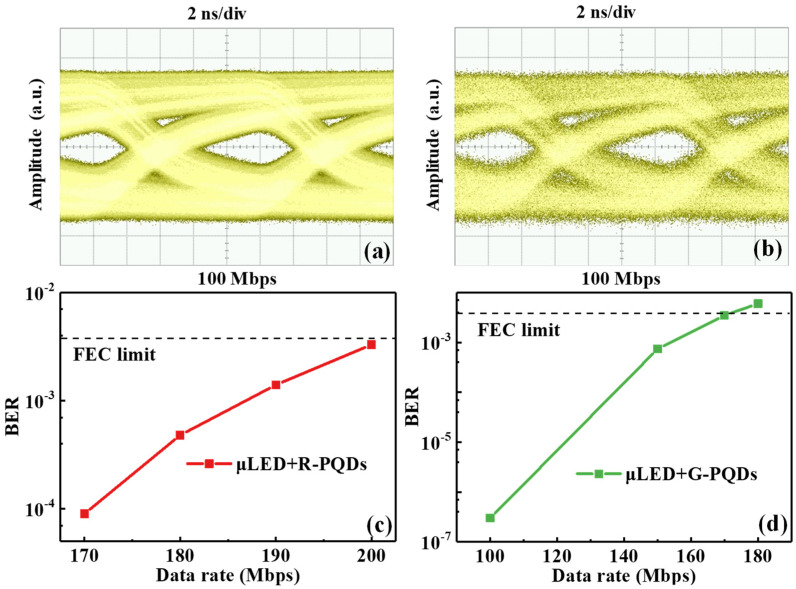
Eye diagrams versus data rates at 100 Mbps of (**a**) μLED + R-PQDs and (**b**) μLED + G-PQDs. BER at different data rates of (**c**) μLED + R-PQDs and (**d**) μLED + G-PQDs. The dashed line shows the FEC threshold.

As shown in Figure 7a,b, the eye diagrams were measured at a data rate of 100 Mbps for μLED + R-PQDs and μLED + G-PQDs, respectively. Attributed to the higher frequency response and signal-to-noise ratio of the R-PQDs, the eye diagram is not only open and clear but also less noisy in comparison with that of the G-PQDs. The maximum data rate of the R-PQDs shown in Figure 7c was achieved at 200 Mbps with a BER of 3.3 × 10^−3^, beneath the forward error correction (FEC) criterion of 3.8 × 10^−3^. Similarly, in Figure 7d, the maximum achievable data rate of the G-PQDs system was 170 Mbps with a BER of 3.5 × 10^−3^, below the FEC threshold. Therefore, both R-PQDs and G-PQDs show excellent potential in optical wireless communication.

To verify high-efficiency illumination and high-speed wireless communication at the same time, the characteristics of the WL-VLC system based on GaN-based white-light LED were further studied. Based on the abovementioned systems, the green light was emitted from the G-PQDs under excitation of the blue light from μLED, and then the mixed light was used to excite the R-PQDs. Consequently, as the blue light, green light, and red light merged, the white light was obtained. The performances of the white-light system were measured and discussed in our further experiments.

**Figure 8:**
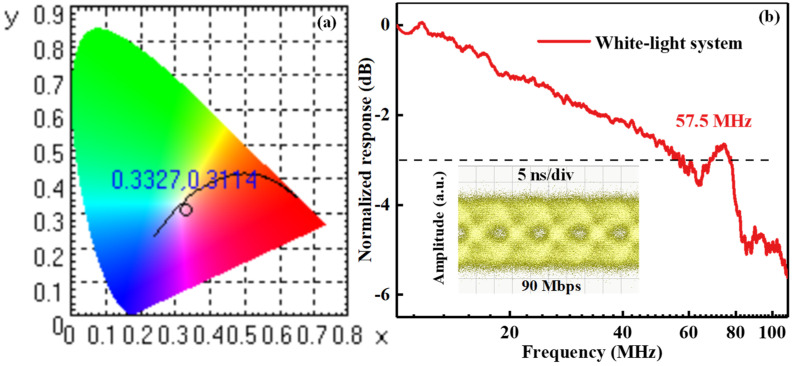
(**a**) CIE color coordinates and (**b**) frequency responses of the white light used in the WL-VLC system. The –3 dB modulation bandwidth is marked by a dashed line. Inset: the eye diagram versus the data rate of the system at 90 Mbps.

As exhibited in Figure 8a, the CIE color coordinates of the WL-VLC system were (0.3327, 0.3114) and the color temperature was 5474 K. Note that compared with the CIE of (0.27, 0.30) in our previous study [38], a better-quality white light of our WL-VLC system was generated, which is very close to the CIE of (0.33, 0.33) for standard white light. As presented in Figure 8b, the maximum bandwidth achieved for the proposed WL-VLC system was 57.5 MHz. The eye diagram of the WL-VLC system at 90 Mbps is shown in the inset of Figure 8b. The above results reveal that the WL-VLC system proposed in this work implemented a high-performance SSL and VLC based on the white-light GaN-based μLED. Compared with the monochromatic light system, the eye diagram is noisy and relatively close. Consequently, substantial efforts will be made to further optimize the InGaN-μLEDs and PQDs to achieve high bandwidth, high light-output power, and better linearity.

## 4. Conclusions

In this study, a WL-VLC system based on white-light GaN-based μLEDs with green and red PQDs as color converters was proposed and demonstrated experimentally for both communication and illumination. The essential optical and electrical performances of monochromatic light from the InGaN-μLEDs, G-PQDs, and R-PQDs, as well as the white light, were studied. The corresponding communication and illumination performances were systematically investigated in detail. We achieved the maximum bandwidths of 162 MHz, 90 MHz, 64 MHz, and 57.5 MHz for InGaN-μLEDs, R-PQDs, G-PQDs, and white-light in our WL-VLC system, respectively. High-quality white light was generated which possessed CIE of (0.3327, 0.3114) and a correlated color temperature of 5474 K. It is worth noting that using R-PQDs and G-PQDs as color converters, the available CIE of (0.3327, 0.3114) is closer to the CIE of (0.33, 0.33) for standard white light, compared with that (0.27, 0.30) of white light using yellow PQDs as color converters in our previous study. These results shed new light on the potential of InGaN-μLEDs combined with PQDs for both high-speed VLC and high-efficiency SSL.

## Data Availability

Data underlying the results presented in this paper are not publicly available at this time, but may be obtained from the authors upon reasonable request. The data that support the findings of this study are available upon reasonable request from the authors.

## Supplementary Materials

The following are available online at https://www.mdpi.com/article/10.3390/nano12040627/s1. Figure S1: (**a**) J–V and (**b**) L–I characteristics of square InGaN-μLEDs with different sizes. Figure S2: The normalized EL spectra of the square μLED with a side length of 80 μm under different applied voltages. Figure S3: The normalized response of the square μLED with a side length of 80 μm under a current of 70 mA. The dashed line represents the forward error correction (FEC) threshold.
