# Supplementary material for: White-Light GaN-μLEDs Employing Green/Red Perovskite Quantum Dots as Color Converters for Visible Light Communication
Source: Nanomaterials (Basel). 2022 Feb 13;12(4):627. doi: 10.3390/nano12040627 (PMC8879407; doi:10.3390/nano12040627)
Supplement: Supplementary file 1 [file nanomaterials-12-00627-s001.zip › nanomaterials-1573619-supplementary.pdf]

nanomaterials

MDPI

Supplementary Material

White-Light GaN-μLEDs Employing Green/Red Perovskite Quantum Dots as Color Converters for Visible Light Communication

Xiaoyan Liu [1], Langyi Tao [1], Shiliang Mei [2], Zhongjie Cui [2], Daqi Shen [1], Zhengxuan Sheng [1], Jinghao Yu [1], Pengfei Ye [1], Ting Zhi [1], Tao Tao [3], Lei Wang [1,*], Ruiqian Guo [2,*] and Pengfei Tian [2,*]

[1] College of Integrated Circuit Science and Engineering, and National and Local Joint Engineering Laboratory for RF Integration and Micro-Packaging Technologies, Nanjing University of Posts and Telecommunications, Jiangsu, Nanjing 210023, China; xiaoyanliu@njupt.edu.cn (X.L.); b18020227@njupt.edu.cn (L.T.); b18020308@njupt.edu.cn (D.S.); b18012128@njupt.edu.cn (Z.S.); 1020021023@njupt.edu.cn (J.Y.); 1320027525@njupt.edu.cn (P.Y.); zhit@njupt.edu.cn (T.Z.)

[2] Institute for Electric Light Sources, School of Information Science and Technology, Fudan University, Shanghai 200433, China; meishiliang@fudan.edu.cn (S.M.); 19110720020@fudan.edu.cn (Z.C.)

[3] Key Laboratory of Advanced Photonic and Electronic Materials, School of Electronic Science and Engineering, Nanjing University, Jiangsu, Nanjing 210046, China; ttao@nju.edu.cn

* Correspondence: leiWang1980@njupt.edu.cn (L.W.); rqguo@fudan.edu.cn (R.G.); pftian@fudan.edu.cn (P.T.)

Citation: Liu, X.; Tao, L.; Mei, S.; Cui, Z.; Shen, D.; Sheng, Z.; Yu, J.; Ye, P.; Zhi, T.; Tao, T.; et al. White-Light GaN-μLEDs Employing Green/Red Perovskite Quantum Dots as Color Converters for Visible Light Communication. *Nanomaterials* **2022**, *12*, 627. https://doi.org/10.3390/nano12040627

Academic Editor(s): Mohammed Jaouad Meziani and Wolfgang Heiss

Received: date: 12 January 2022

Accepted: date: 09 February 2022

Published: 13 February 2022

Publisher's Note: MDPI stays neutral with regard to jurisdictional claims in published maps and institutional affiliations.

Copyright: © 2022 by the authors. Licensee MDPI, Basel, Switzerland. This article is an open access article distributed under the terms and conditions of the Creative Commons Attribution (CC BY) license (https://creativecommons.org/licenses/by/4.0/).

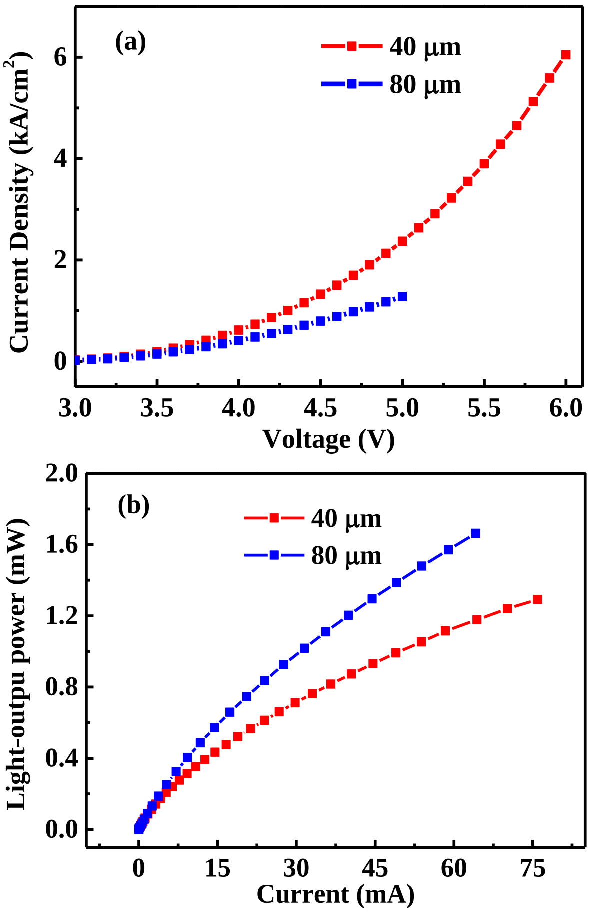

Figure S1. (**a**) J–V and (**b**) L–I characteristics of the square InGaN-μLEDs with different sizes.

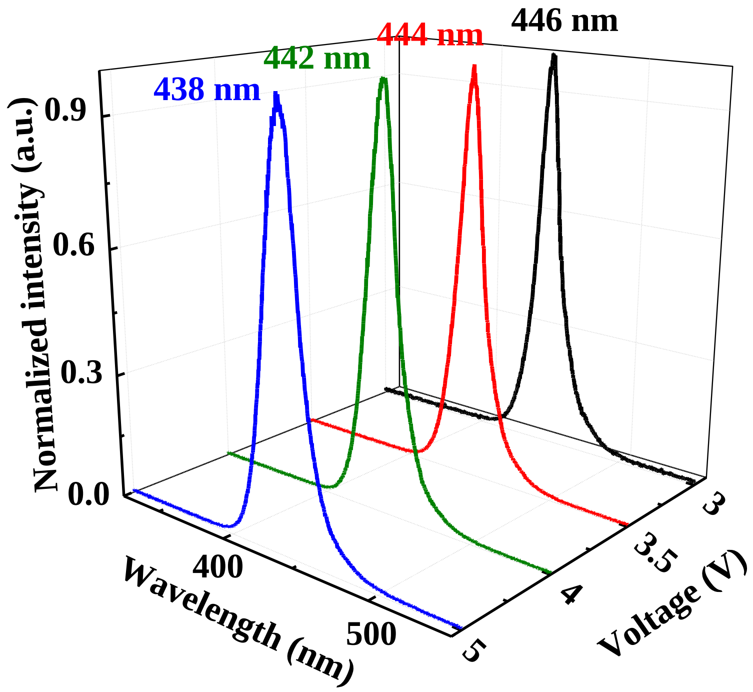

Figure S2. The normalized EL spectra of the square μLED with a side length of 80 μm under different applied voltages from 3 V to 5 V.

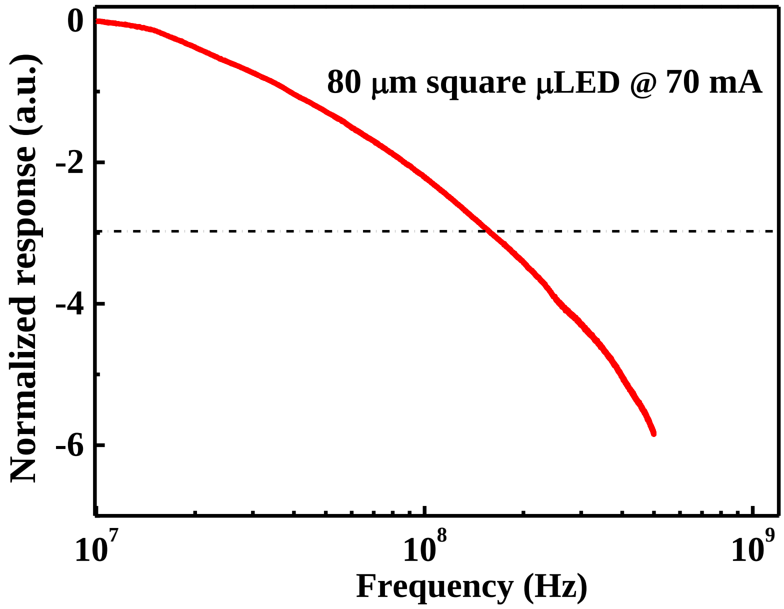

Figure S3. The normalized response of the square μLED with a side length of 80 μm under a current of 70 mA. The dashed line is to label the −3 dB bandwidth.
